# Probabilistic Encoding Models for Multivariate Neural Data

**DOI:** 10.3389/fncir.2019.00001

**Published:** 2019-01-28

**Authors:** Marcus A. Triplett, Geoffrey J. Goodhill

**Affiliations:** Queensland Brain Institute and School of Mathematics and Physics, The University of Queensland, Brisbane, QLD, Australia

**Keywords:** neural coding, calcium imaging, population code, brain-computer interfaces, generalized linear model, Gaussian process, factor analysis

## Abstract

A key problem in systems neuroscience is to characterize how populations of neurons encode information in their patterns of activity. An understanding of the encoding process is essential both for gaining insight into the origins of perception and for the development of brain-computer interfaces. However, this characterization is complicated by the highly variable nature of neural responses, and thus usually requires probabilistic methods for analysis. Drawing on techniques from statistical modeling and machine learning, we review recent methods for extracting important variables that quantitatively describe how sensory information is encoded in neural activity. In particular, we discuss methods for estimating receptive fields, modeling neural population dynamics, and inferring low dimensional latent structure from a population of neurons, in the context of both electrophysiology and calcium imaging data.

## 1. Introduction

An animal's perceptual capabilities critically depend on the ability of its brain to form appropriate representations of sensory stimuli. However, the neural activity induced by a specific stimulus is highly variable, suggesting that neural encoding is a fundamentally probabilistic process. Characterizing the neural code thus requires statistical methods for relating stimuli to distributions of evoked patterns of activity. Modern techniques for recording such neural activity include multi-electrode arrays, which provide access to the behavior of populations of neurons at millisecond resolution, and optical imaging with genetically encoded calcium (Chen et al., 2013) and voltage indicators (Abdelfattah et al., 2018), which allow thousands of neurons to be recorded simultaneously (Ahrens et al., 2013; Chen et al., 2018). However, while improvements in multi-neuron recording allow us to probe neural circuits in great detail, they are accompanied by a need for computational techniques that scale to entire neural populations.

A statistical model for neural coding describes how a stimulus is mathematically related to a pattern of neural activity. By fitting the model one can extract important variables that quantitatively describe the encoding procedure taking place. For instance, such models enable the estimation of receptive fields and/or interneuronal coupling strengths. In contrast to other methods for inferring these variables, an approach based on statistical models situates the task of estimating salient parameters in a coherent mathematical framework, often with proof of asymptotic optimality or computational efficiency. By making explicit assumptions about how the data was generated, statistically principled approaches are often capable of identifying patterns in neural data which are challenging to find with simpler methods.

Linear and generalized linear models are among the most straightforward classes of statistical models for spike trains and assume that a neuron's activity is a noisy linear combination of the stimulus features. These models are highly effective at explaining the structure of sensory receptive fields and are computationally tractable, but do not explicitly model the temporal structure of the recorded signal and have difficulty accounting for correlations between neurons in short time windows. An important aspect of these correlations is their tendency to be modular, with distinct groups of neurons showing cofluctuating activity. Latent factor models attempt to uncover the low dimensional structure that gives rise to this correlated variability, and recent efforts have focused on extracting low dimensional structure that evolves smoothly through time using a latent linear dynamical system or Gaussian process (Cunningham and Byron, 2014).

A further challenge is presented by calcium imaging, which provides only indirect access to neural activity through recorded fluorescence levels that reflect the concentration of calcium within a neuron. Often this data can be more difficult to interpret than electrophysiological recordings as there are a number of biophysical stages between stimulus presentation and fluorescence imaging where noise can enter and information can be lost. Using a generative model for calcium imaging data, however, one can explicitly account for the process through which action potentials are transformed into fluorescence levels. Fitting the generative model amounts to deconvolving the fluorescence signal to estimate the underlying spike train timeseries, and conventional encoding models can then be applied to deconvolved data. However, the ability to obtain spike counts from fluorescence data is highly constrained by experimental conditions, which motivates the development of encoding models specific to calcium imaging that do not necessarily involve spike train deconvolution.

While previous reviews have focused on estimating stimulus-response functions (Paninski et al., 2007; Pillow, 2007; Meyer et al., 2017), neural decoding (Paninski et al., 2007; Quiroga and Panzeri, 2009), and conceptual overviews of models and data analysis techniques (Cunningham and Byron, 2014; Paninski and Cunningham, 2018), this review instead discusses a range of recent exemplary models and their successful application to experimental data. Our goal is to provide sufficient mathematical detail to appreciate the respective strengths and weaknesses of each model, while leaving formal treatment of their associated fitting algorithms to their original sources.

## 2. Linear and Generalized Linear Models

We first briefly review now-standard material on models for single-neuron spike trains, primarily to develop the theory, terminology, and notation necessary for more recent work focused on multivariate models.

### 2.1. The Linear-Gaussian Model

**Figure 1:**
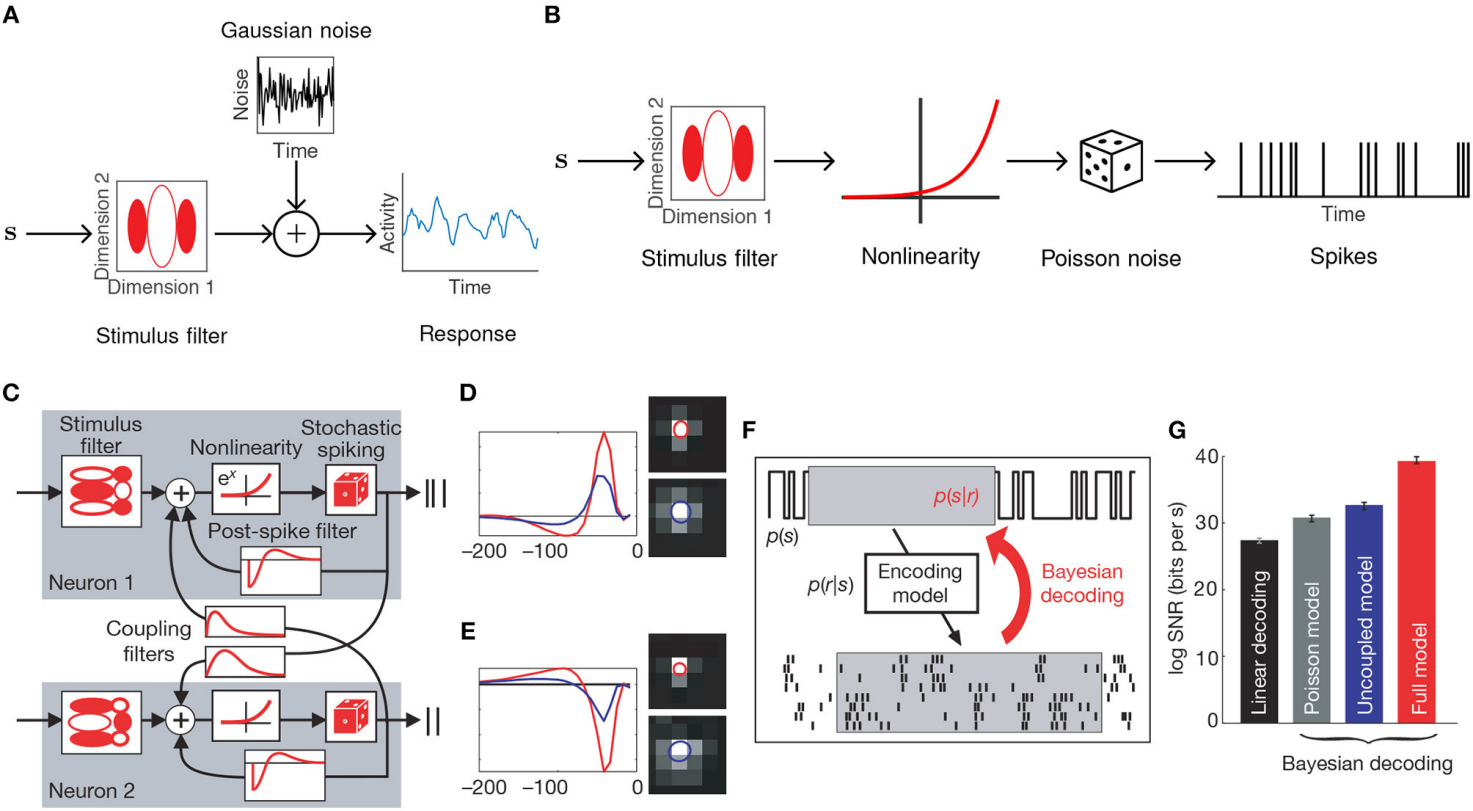
Generalized linear encoding models. **(A)** Basic linear-Gaussian model. A neuron's response is modeled as a linear combination of the stimulus components with additive Gaussian noise. In this example, the stimulus filter represents a two-dimensional visual receptive field. **(B)** The LNP model extends the linear-Gaussian model with a static nonlinearity applied to the filtered stimulus, together with Poisson observations to directly model spike counts. **(C)** Multi-neuron GLM encoding model used in Pillow et al. (2008). **(D)** Center (red) and surround (blue) components of temporal (left) and spatial (right) stimulus filters for the GLM fitted to an example ON retinal ganglion cell. **(E)** Same as **(D)**, but for an example OFF cell. In contrast to the ON cell, the OFF cell has an inhibitory effect on spiking. **(F)** Schematic of Bayesian decoding process. The encoding model *p*(**r**|**s**) is first fit by maximum likelihood. Then stimuli are decoded as the mean of the posterior stimulus distribution *p*(**s**|**r**) obtained by Bayes rule. **(G)** Performance of various decoders. Decoding using a GLM with interneuronal coupling filters (full model) substantially increases performance over models that do not account for interaction effects (linear, Poisson, and uncoupled models). **(C–G)** Adapted with permission from Pillow et al. (2008).

Among the simplest probabilistic models for a neuron's response *r* to a stimulus vector **s** is the linear-Gaussian model (Figure 1A), which assumes that a neuron linearly filters the features of **s** as

(1) $$\begin{array}{r} r={\text{w}}^{⊤}\text{s}+\epsilon ,\;\epsilon \sim \;N\left(0,\sigma^{2}\right) \end{array}$$

where the vector **w** is the stimulus filter, ϵ is an additive noise variable, and $N\left(0,\sigma^{2}\right)$ is a Gaussian distribution with mean 0 and variance σ^2^ (see Table 1 for a table of notation). In the case of visual processing the stimulus **s** is a vector of pixel intensities for each point in the visual field, the stimulus filter **w** corresponds to the classical visual receptive field, and the response *r* is either the spike count or firing rate within some time window following the stimulus. Assuming stimuli **s**_1_, …, **s**_*K*_ are presented over *K* trials yielding responses *r*_1_, …, *r*_*K*_ with independent and identically distributed noise as in Equation 1, the maximum likelihood estimate (MLE, see Table 2 for a table of abbreviations) for the filter **w** is given by

(2) $$\begin{array}{r} \hat{\text{w}}=\underset{\text{w}}{\mathrm{argmax}}\overset{K}{\underset{k=1}{\prod }}p\left(r_{k}|{\text{s}}_{k},\text{w}\right). \end{array}$$

Since the noise model is Gaussian, the solution to Equation (2) is simply the ordinary least squares solution (Bishop, 2006)

(3) $$\begin{array}{r} \hat{\text{w}}={\left({\text{S}}^{⊤}\text{S}\right)}^{-1}{\text{S}}^{⊤}\text{r} \end{array}$$

where $\text{S}={\left({\text{s}}_{1},\ldots ,{\text{s}}_{K}\right)}^{⊤}$ is the stimulus design matrix and $\text{r}={\left(r_{1},\ldots ,r_{K}\right)}^{⊤}$ is the vector of neuron responses.

**Table 1:** Table of notation.

**Symbol**	**Parameter**
*r*	Response
**s**	Stimulus
**w**	Stimulus filter
**S**	Stimulus design matrix
λ	Intensity, mean
ϵ, ξ, ξ_*h*_ℓ__	Gaussian noise variable
σ, σ_*s*_, ν	Noise standard deviation
**μ**, **μ**_*s*_	Baseline activity, mean
**h**	Spike history vector
**J**_*ij*_	Spike history coupling filter
**x**	Latent factor
**Ψ**_*s*_	Diagonal variance matrix
**Λ**, **Λ**_*s*_	Factor loading matrix
**Σ**_*s*_	Covariance matrix
τ	Gaussian process timescale
*n*	Spike count
*c*	Calcium concentration
γ	Autoregressive coefficient
α	Fluorescence scale
β	Baseline fluorescence level
*f*	Fluorescence level
*p*	Spike probability
*h*_ℓ_	Refractory term
*k*_*d*_	Dissociation constant
N	Gaussian distribution
Pois	Poisson distribution
Bern	Bernoulli distribution

*A subscripted s indicates stimulus-specific parameters*.

**Table 2:** Table of abbreviations.

**Abbreviation**	**Meaning**
MLE	Maximum likelihood estimate
STA	Spike-triggered average
LNP	Linear-nonlinear-Poisson
GLM	Generalized linear model
FA	Factor analysis
EM	Expectation maximization
GP	Gaussian process
GPFA	Gaussian process factor analysis
PLDS	Poisson linear dynamical system

A common interpretation of the estimator in Equation (3) is in terms of the spike-triggered average (STA) of the stimulus, which is the filter obtained by averaging over the stimuli that elicited a response,

(4) $$\begin{array}{r} {\hat{\text{w}}}_{\text{STA}}=\frac{1}{N}{\text{S}}^{⊤}\text{r} \end{array}$$

where *N* is the total number of spikes. When the stimulus ensemble follows a multivariate Gaussian with independent dimensions (and is therefore not biased toward any particular region of the feature space) the STA is the optimal filter (Chichilnisky, 2001; Dayan and Abbott, 2001; Simoncelli et al., 2004) and is proportional to the MLE. In general, the MLE pre-multiplies the STA by the inverse of the autocorrelation matrix **S**^⊤^**S** of the stimulus ensemble to correct for bias in the presented stimuli, and thus corresponds to a whitened STA. Further discussion of the STA and its connection to the MLE can be found in Simoncelli et al. (2004) and Meyer et al. (2017).

### 2.2. The Linear-Nonlinear-Poisson Model

While a linear model can recover basic receptive field structure, it fails to capture the nonlinear changes in firing rate observed in electrophysiological recordings in cortex. In addition, the assumption of Gaussian noise leads to continuous (and possibly negative) estimates of spike counts. The linear-nonlinear-Poisson (LNP) model addresses these shortcomings by equipping the generative model with a static nonlinearity following the linear filtering, and a Poisson noise model to directly model the number of spikes generated within a fixed time-window (Figure 1B) (Chichilnisky, 2001). Let *t* = 1, …, *T* index over time bins. The LNP model assumes spikes follow an inhomogeneous Poisson process with time-varying firing rate λ(*t*),

(5) $$\begin{array}{r} \lambda \left(t\right)=g\left({\text{w}}^{⊤}\text{s}\left(t\right)\right),\;r\left(t\right)\sim \text{Pois}\left(\lambda \left(t\right)\right) \end{array}$$

where *g* is a nonlinear activation function. While this nonlinearity can be estimated nonparametrically for each neuron (Simoncelli et al., 2004), it is often chosen to be *g*(*x*) = exp(*x*) as this ensures a non-negative intensity λ and tractable model fitting. Note that the specified firing rate λ(*t*) will depend on the width Δ of the time bins or imaging rate, but for clarity here and for the remainder of the paper we omit explicit dependence of λ(*t*) on Δ.

Assuming *g*(*x*) = exp(*x*) and that the responses *r*(*t*) are count data, the MLE for the LNP model is the solution

(6) $$\begin{array}{r} \hat{\text{w}}=\underset{\text{w}}{\mathrm{argmax}}\overset{T}{\underset{t=1}{\prod }}p\left(r\left(t\right)|\text{s}\left(t\right),\text{w}\right)=\underset{\text{w}}{\mathrm{argmax}}\overset{T}{\underset{t=1}{\sum }}\left(r\left(t\right)\ln\;\lambda \left(t\right)-\lambda \left(t\right)\right) \end{array}$$

where the second equality follows by substituting the Poisson mass function and taking logarithms. The LNP model can be fit by standard gradient-based optimization methods since the intensity function λ(*t*) is differentiable with respect to the filter parameters **w** and the log-likelihood function is concave (Paninski, 2004).

Regularization is a commonly used technique in machine learning for preventing a model from overfitting the training data. When maximizing the log-likelihood function for the LNP model with regularization, one penalizes the filter components whenever they deviate from zero

(7) $$\begin{array}{r} \hat{\text{w}}=\underset{\text{w}}{\mathrm{argmax}}\overset{T}{\underset{t=1}{\sum }}\left(r\left(t\right)\ln\;\lambda \left(t\right)-\lambda \left(t\right)\right)-\eta ||\text{w}|{|}_{p} \end{array}$$

where || · ||_*p*_ denotes the *L*_*p*_ norm and η > 0 is a penalty coefficient. Setting *p* = 1 or *p* = 2 corresponds, respectively, to LASSO and ridge regression (Friedman et al., 2001), encouraging a sparse filter **w**. Maximizing the penalized log-likelihood is equivalent to performing posterior inference in a Bayesian regression model where **w** has a Laplacian (for *p* = 1) or Gaussian (for *p* = 2) prior (Wu et al., 2006). In many circumstances, such as when the data exhibits high noise levels, the ordinary (unpenalized) MLE cannot recover realistic receptive fields and needs to be constrained by regularization or priors (Sahani and Linden, 2003). Such Bayesian methods become highly effective in regimes of high noise, and a number of Bayesian extensions of receptive field inference invoke more subtle machine learning methods. For example, automatic relevance determination (Sahani and Linden, 2003) places a Gaussian prior on each element **w**_*i*_ of the filter and iteratively updates the prior variance until the filter components corresponding to irrelevant stimulus features effectively vanish from the model. Automatic locality determination, on the other hand, involves constructing receptive field priors encoding the information that receptive fields tend to be localized in space, time relative to the stimulus, and spatiotemporal frequency (Park and Pillow, 2011).

### 2.3. Extensions of the LNP Model

The LNP model is a special case of a generalized linear model (GLM): a class of encoding models that generalize the simple linear-Gaussian model to models that follow linear filtering with a static nonlinearity and any noise model from the exponential family. While there is in general no probability mass function for a multivariate extension of the Poisson distribution, the GLM framework allows one to incorporate interaction effects between different neurons, thereby allowing statistical models for single neurons to be used for entire populations. The LNP model is extended by the addition of spike-history filters **J**_*ij*_ for all pairs of neurons *i* and *j*, intended to capture refractory effects for individual neurons (i.e., when *i* = *j*) and interaction effects between neurons (*i* ≠ *j*), giving

(8) $$\begin{array}{r} \lambda_{i}\left(t\right)=\exp\left({\text{w}}_{i}^{⊤}\text{s}\left(t\right)+\overset{N}{\underset{j=1}{\sum }}{\text{J}}_{ij}^{⊤}{\text{h}}_{j}\left(t\right)\right),\;r_{i}\left(t\right)\sim \text{Pois}\left(\lambda_{i}\left(t\right)\right) \end{array}$$

where **w**_*i*_ is the stimulus filter for neuron *i*, ${\text{h}}_{j}\left(t\right)={\left(r_{j}\left(t-1\right),\ldots ,r_{j}\left(t-\tau \right)\right)}^{⊤}$ is a vector of neuron *j*'s spike history, and τ determines the length of the spike history window. The addition of the coupling filters allows the GLM to model the correlation structure within a population of neurons, as opposed to a model consisting of independent LNP neurons. Note, however, that the GLM is only well defined for coupling filters that act on the recent spike history of other neurons within the population, and cannot model correlations that arise from coactivity with zero time-lag (Macke et al., 2011). This motivates the use of latent variable models (see below), where simultaneous correlations arise among neurons whose activity is concurrently modulated by a shared factor.

Nonetheless, the GLM has been successfully applied to many data sets (Pillow et al., 2005, 2008; Park et al., 2014). Notably, Pillow et al. (2008) applied the GLM to a population of retinal ganglion cells from the fly (Figures 1C–E), obtained a complete characterization of the network's spatiotemporal correlation structure, and showed how incorporating these correlations yields a ~20% increase in estimated information about the presented visual scene (Figures 1F,G).

## 3. Latent Factor Models

### 3.1. Encoding With Factor Analysers

A frequent observation when recording population responses to the repeated presentation of identical stimuli is that variability tends to be correlated among groups of neurons. Such correlated variability (also known as shared variability or noise correlations) can substantially impact the efficacy of a neural code depending on the particular correlation structure (Abbott and Dayan, 1999; Schneidman et al., 2006; Lin et al., 2015), and suggests that there may be factors present that comodulate the responses of groups of neurons. Factor analysis (FA), a probabilistic generalization of principal components analysis, is a classical model for inferring the latent group structure that can give rise to correlated variability.

In a Gaussian coding scheme with independent neurons, a population response **r** to a fixed stimulus *s* has a probability density given by

(9) $$\begin{array}{r} p\left(\text{r}|s\right)=N\left(\text{r}|\mu_{s},\sigma_{s}^{2}{\text{I}}_{N}\right) \end{array}$$

where the vector ***μ***_*s*_ is the mean population response, $\sigma_{s}^{2}$ is a noise variance common to each neuron, and **I**_*N*_ is the *N* × *N* identity matrix. While this model is analytically tractable with closed-form expressions for **μ**_*s*_ and σ_*s*_, the diagonal covariance matrix means it fails to account for the correlation structure that may be present in the data. As shown in e.g., Pillow et al. (2008), this additional information can considerably influence decoding accuracy.

On the other hand, a Gaussian model with an unconstrained covariance matrix **Σ**_*s*_ yields a density of the form

(10) $$\begin{array}{r} p\left(\text{r}|s\right)=N\left(\text{r}|\mu_{s},{\text{Σ}}_{s}\right), \end{array}$$

which, in principle, could outperform the Gaussian version that uses an unrealistic assumption of independently acting neurons (Santhanam et al., 2009). However, the covariance matrix **Σ**_*s*_ has (*N*^2^ + *N*)/2 parameters to be learned per stimulus, requiring an amount of data that is impractically large to obtain experimentally for large *N*.

FA is a more moderate approach that attempts to capture shared variability in population activity by specifying a tractable parameterization of the covariance matrix. For FA the covariance matrix is defined as ${\text{Σ}}_{s}={\text{Λ}}_{s}{\text{Λ}}_{s}^{⊤}+{\text{Ψ}}_{s}$, where ${\text{Ψ}}_{s}\in {\mathbb{R}}^{N\times N}$ is a diagonal matrix, ${\text{Λ}}_{s}\in {\mathbb{R}}^{N\times q}$ is a factor loading matrix (analogous to the component loading matrix in principal components analysis), and *q* < *N* determines the rank of ${\text{Λ}}_{s}{\text{Λ}}_{s}^{⊤}$. Hence the population response **r** is distributed as

(11) $$\begin{array}{r} p\left(\text{r}|s\right)=N\left(\text{r}|\mu_{s},{\text{Λ}}_{s}{\text{Λ}}_{s}^{⊤}+{\text{Ψ}}_{s}\right). \end{array}$$

This decomposes **Σ**_*s*_ into two matrices that capture separate aspects of the response variability: ${\text{Λ}}_{s}{\text{Λ}}_{s}^{⊤}$ is a low-rank matrix that captures the variability that is shared across neurons, whereas the diagonal matrix **Ψ**_*s*_ captures variability private to each neuron (Churchland et al., 2010). A critical observation is that the FA covariance matrix only requires (*q* + 1)*N* parameters, which is less than (*N*^2^ + *N*)/2 whenever *q* < (*N* − 1)/2. Since *q* is usually chosen to be small, the FA covariance matrix requires much fewer parameters to be learned from the data.

An equivalent formulation of FA models the population response to a stimulus *s* as the projection from a low dimensional space of latent factors into the N-dimensional population space. This low dimensionality constraint forces any variability that the latent factors account for to be shared across groups of neurons, which leads to a modular correlation structure in the population recording. The generative model for the population response **r**_*s*_ given a stimulus *s* is

(12) $$\begin{array}{rc} {\text{r}}_{s} & ={\text{Λ}}_{s}{\text{x}}_{s}+\mu_{s}+{\text{ϵ}}_{s} \end{array}$$

(13) $$\begin{array}{rc} {\text{x}}_{s} & \sim \;N\left(0,{\text{I}}_{q}\right) \end{array}$$

(14) $$\begin{array}{rc} {\text{ϵ}}_{s} & \sim \;N\left(0,{\text{Ψ}}_{s}\right), \end{array}$$

where ${\text{x}}_{s}\in {\mathbb{R}}^{q}$ denotes the vector of latent factors, which are assumed to be independent with a Gaussian prior. These factors are intended to reflect unobserved brain states and could be physiologically realized as, e.g., shared gain modulation by downstream circuits. Note that the formulation of FA in Equation (11) can be recovered from Equations (12–14) by marginalizing over the latent factors.

Maximum likelihood estimation of the FA parameters θ_*s*_ = (***μ***_*s*_, **Ψ**_*s*_, **Λ**_*s*_, σ_*s*_) is complicated by the presence of latent variables **x**, as the MLE ${\hat{\theta }}_{s}$ depends on an estimated $\hat{\text{x}}$, and vice versa. FA thus uses the Expectation Maximization (EM) algorithm, an iterative procedure for fitting latent variable models (Dempster et al., 1977; Ghahramani et al., 1996). One must also choose the dimensionality *q* of the latent space, typically with a standard model selection procedure such as a comparison of the cross-validated log-likelihood or with an information criterion (Schwarz, 1978).

The FA method was applied to rhesus monkeys with brain-computer interfaces implanted in area PMd (Santhanam et al., 2009). Monkeys were trained on reaching tasks and the authors attempted to infer the intended target from electrophysiological data using a decoder based on the FA encoding model. By fitting the factor analyser, the decoder inferred the latent factors that comodulated neurons' responses. Incorporating this information led to substantial improvements in decoding accuracy over decoders based on independent Gaussian and Poisson encoding models.

### 3.2. Gaussian Process Factor Analysis

The peristimulus time histogram averages spike trains over many trials to robustly estimate the aggregate effect of presenting a stimulus. Similarly, the FA encoding model is fit by pooling responses across trials to estimate the parameters θ_*s*_. While this across-trial synthesis is necessary for fitting model parameters accurately, it will fail to reveal possibly important subtleties in neural activity within individual trials (Churchland et al., 2007, 2010; Afshar et al., 2011).

**Figure 2:**
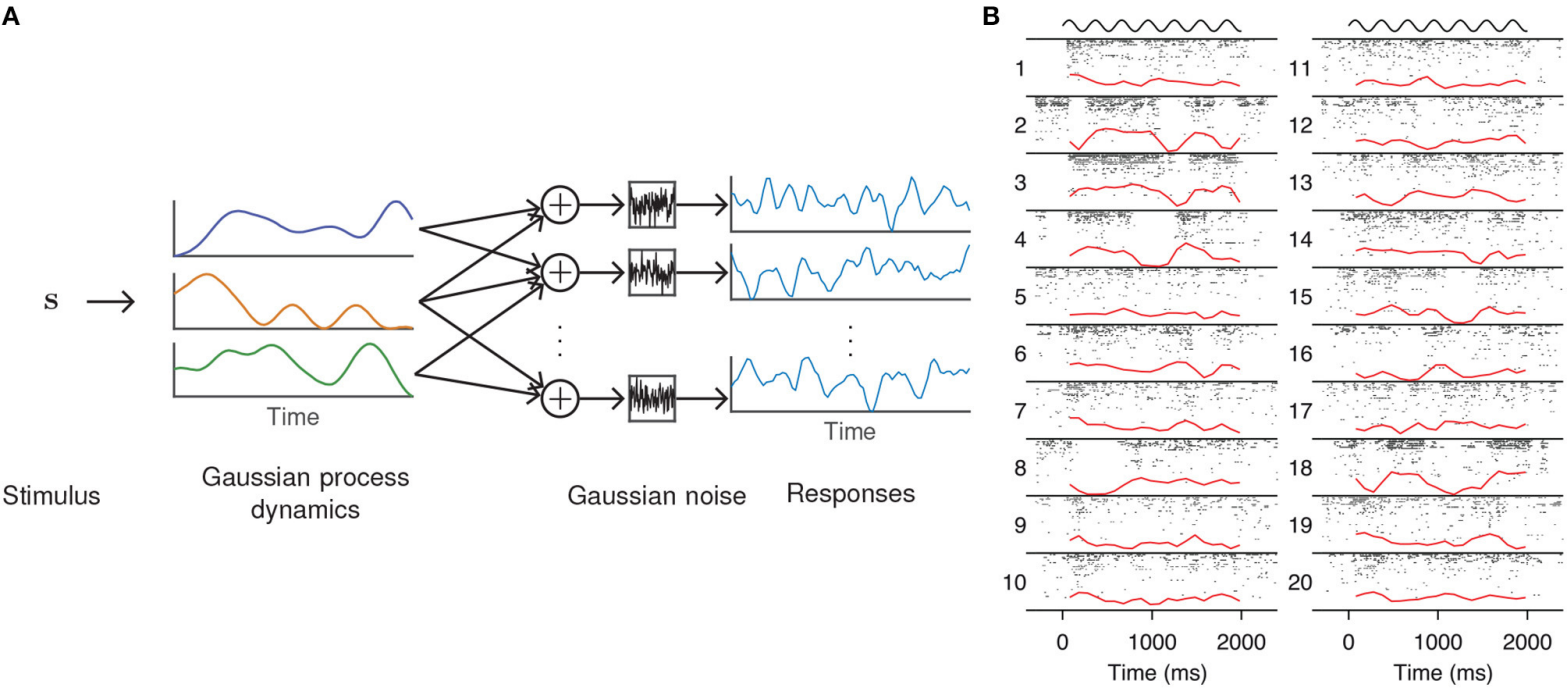
GPFA model of neural population activity. **(A)** Schematic of the GPFA model, which adapts FA by including a GP prior on the evolution of the latent factors. Colored lines above label “Gaussian process dynamics” represent individual latent factors evolving smoothly through time. Each neuron's firing rate is obtained by linearly combining the latent factors at each time point. **(B)** Inferred latent factors from 20 trials of population recordings from anesthetized macaque primary visual cortex. Each recording (indexed by numbers to the left of each column) was best explained by a single factor (red curves) that evolved independently of the stimulus (black curves above each column). At high firing rates, this single factor explained as much as 40% of the variance of individual neuron activity. Panel adapted with permission from Ecker et al. (2014).

One way to adapt FA to single-trial analysis is to model the temporal evolution of the latent factors. A common technique in machine learning for enforcing temporal structure (or smoothness more generally) is Gaussian process (GP) regression, a Bayesian technique for nonparametric statistical modeling that places a GP prior on the latent variables (Williams and Rasmussen, 2006). The Gaussian process factor analysis (GPFA, Figure 2A) model (Yu et al., 2009) defines a GP for each dimension of the latent state ℓ = 1, …, *q*, which, in the case of discretely indexed time, reduces to a collection of multivariate Gaussians

(15) $$\begin{array}{rc} {\text{x}}^{\left(\ell \right)} & \sim \;N\left(0,\text{K}\right). \end{array}$$

Here each **x**^(ℓ)^ = (**x**^(ℓ)^(1), …, **x**^(ℓ)^(*T*))^⊤^. Elements of the covariance matrix **K** are typically determined by the squared exponential kernel for encouraging smoothness

(16) $$\begin{array}{r} {\text{K}}_{t_{1},t_{2}}=\sigma_{f}^{2}\exp\left(-\frac{{\left(t_{1}-t_{2}\right)}^{2}}{2\tau^{2}}\right)+\sigma_{n}^{2}\delta \left(t_{1},t_{2}\right) \end{array}$$

where δ is the Kronecker delta function and σ_*f*_ and σ_*n*_ are parameters controlling the variance of the GP. The observed responses are then modeled as in FA,

(17) $$\begin{array}{rc} \text{r}\left(t\right) & =\text{Λ}\text{x}\left(t\right)+\mu +\text{ϵ}\left(t\right) \end{array}$$

(18) $$\begin{array}{rc} \text{ϵ}\left(t\right) & \sim \;N\left(0,\text{Ψ}\right) \end{array}$$

where **x**(*t*) is the latent state at time *t*, **Λ** is the factor loading matrix, and ***μ*** is a baseline activity level. GPFA can be viewed as a sequence of factor analysers (one for each time point) whose dimensions are linked together by smooth GPs. Note that while we have specified a single GP timescale τ, one can also assign distinct timescales τ_*i*_ to each dimension at the cost of an increase in computational overhead.

An advantage of GPFA is that the posterior over latent states **x**^(ℓ)^ can be written down analytically because both the prior and likelihood are Gaussian, which form a conjugate pair (Bishop, 2006). This naturally leads to model fitting with the EM algorithm, where the updates for the parameter estimates are analogous to EM for FA (Ghahramani et al., 1996; Yu et al., 2009). Other examples of GP-based latent factor models are given in Nam (2015), Zhao and Park (2017), and Wu et al. (2017).

In a study of opioid anesthesia in macaque primary visual cortex, Ecker et al. (2014) used GPFA to investigate stimulus-driven patterns of population activity. The fitted model possessed a single latent dimension that unmasked spontaneous transitions between periods of inactivity and highly elevated activity (Figure 2B). This single factor explained the observed increase in noise correlations and accounted for 40% of the variance of individual neuron firing rates. The extracted latent factors spanned a range of timescales, with some data best described by a latent factor whose strength changed slowly, on the order of several minutes. Similar up and down states had previously been seen only with non-opioid anesthetics.

### 3.3. The Poisson Linear Dynamical System

**Figure 3:**
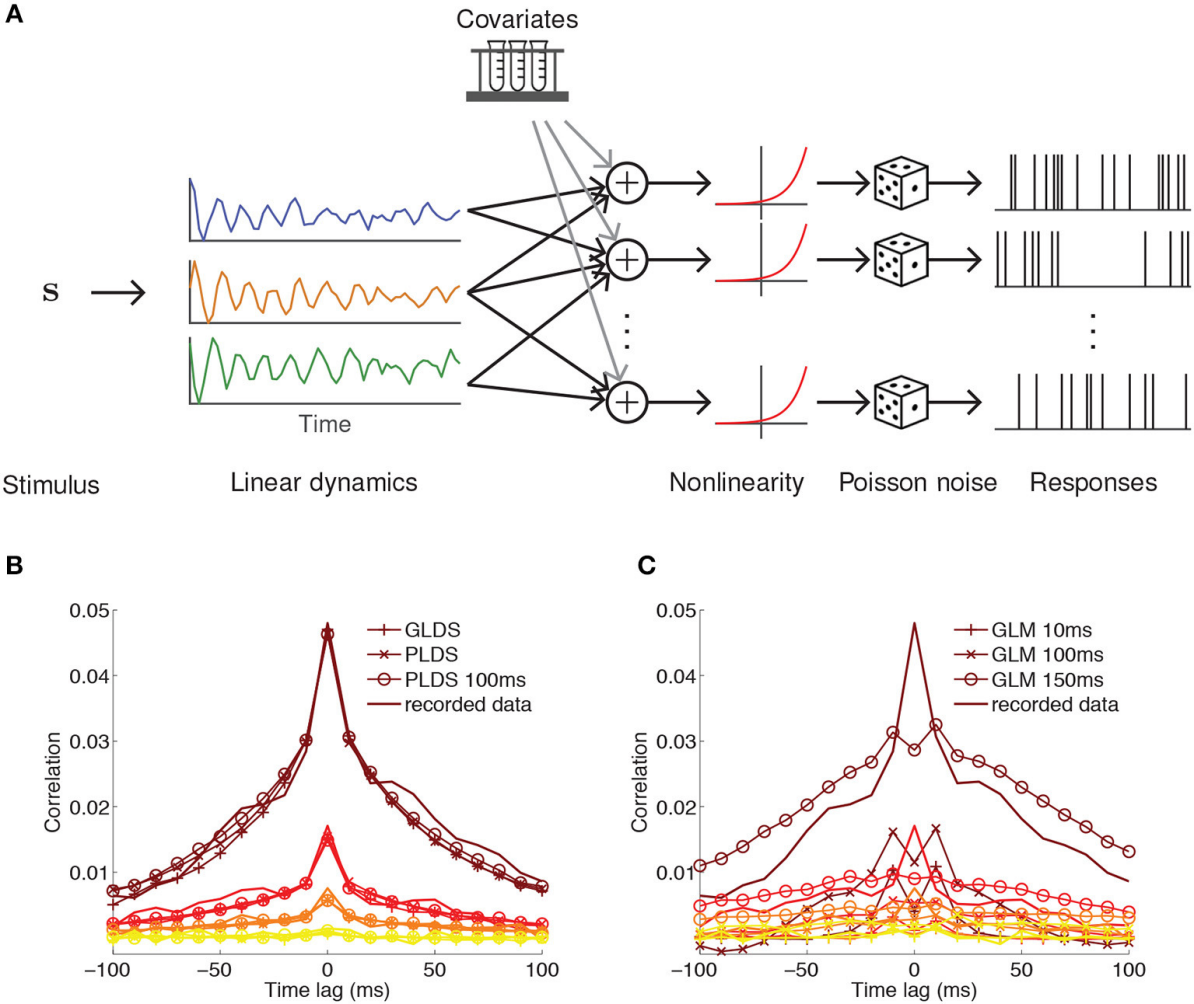
PLDS model of neural population activity. **(A)** Schematic of the PLDS model. Analogous to GPFA, the PLDS model places a linear dynamical system prior over the latent factors. The activity of the factors is combined linearly, rectified by a nonlinearity, and determines Poisson spiking intensity for each neuron. Experimental parameters, spike history, and gain variables are often incorporated as covariates in the linear combination stage. **(B)** Averaged cross-correlations for latent dynamical system models with Gaussian observations (GLDS), Poisson observations (PLDS), and Poisson observations with spike history dependence (PLDS 100 ms). Groups of colored lines represent the average cross-correlation for the most correlated group of neurons (top group, brown) to the least correlated group (bottom group, yellow). Latent dynamical systems models have cross-correlations that align closely with the recorded data. **(C)** Same as **(B)**, but for GLMs with spike history filters of varying duration. Misalignment between the cross-correlations obtained from the model and the recorded data indicate that GLMs struggle to account for correlations at short time lags, in contrast to latent factor models where they arise naturally. **(B,C)** Adapted from Macke et al. (2011).

An alternative approach for latent trajectory modeling is to estimate the underlying linear dynamics of the latent state (Macke et al., 2011; Churchland et al., 2012; Pandarinath et al., 2018a). While the classical Kalman filter is the most thoroughly developed method for estimating the transition matrix in a linear dynamical system, a more appropriate generative model for neurons is the Poisson linear dynamical system (PLDS, Figure 3A) (Macke et al., 2011), which substitutes Poisson observations for the Gaussian emissions in the Kalman filter to directly model observed spike counts. The latent state ${\text{x}}_{k}\left(t\right)\in {\mathbb{R}}^{q}$ on trial *k* at time bin *t* follows linear Markovian dynamics

(19) $$\begin{array}{rc} {\text{x}}_{k}\left(t+1\right) & =\text{A}{\text{x}}_{k}\left(t\right)+\text{b}\left(t\right)+{\text{ϵ}}_{k}\left(t+1\right) \end{array}$$

(20) $$\begin{array}{rc} {\text{x}}_{k}\left(1\right) & \sim \;N\left(0,{\text{Q}}_{1}\right) \end{array}$$

(21) $$\begin{array}{rc} {\text{ϵ}}_{k}\left(t\right) & \sim \;N\left(0,\text{Q}\right) \end{array}$$

where **A** is the dynamics matrix, **Q** is the noise covariance for the latent linear dynamics, and **Q**_1_ is the covariance of the initial state. The latent dynamics are driven by a variable **b**(*t*) that captures stimulus-specific effects. Note that the PLDS model is formulated with explicit dependence on the trial index *k*, so that **b**(*t*) accounts for stimulus effects that are trial-independent. Similar to the LNP model, the observed spike responses on trial *k* then follow a Poisson distribution with mean λ_*i,k*_(*t*) derived from the latent state. For neuron *i* this takes the form

(22) $$\begin{array}{r} \lambda_{i,k}\left(t\right)=g\left({\text{Λ}}_{\left(i\right)}{\text{x}}_{k}\left(t\right)+\mu_{i}\right),\;r_{i,k}\left(t\right)\sim \text{Pois}\left(\lambda_{i,k}\left(t\right)\right). \end{array}$$

Here the latent state influences an individual neuron *i* according to a row **Λ**_(*i*)_ of the factor loading matrix **Λ**, and the low dimensionality of the latent state leads to the correlated variability as in the discussion of FA. Common choices for the nonlinearity include *g*(*x*) = exp(*x*) (Macke et al., 2011) and *g*(*x*) = ln(1 + exp(*x*)) (Buesing et al., 2017).

This model can be modified in various ways to suit the data. For example, the stimulus drive term **b** in Equation (19) can be moved within the nonlinearity in Equation (22), so that the latent dynamics are decoupled from the stimulus and only reflect changes internal to the brain. The intensity can be further extended by adding terms for, e.g., multiplicative gain (Buesing et al., 2017) and spike history (Macke et al., 2011) to capture refractory effects. A major advantage of latent factor models is their ability to account for correlations within short time intervals (Figure 3B), which GLMs struggle to match (Figure 3C).

The PLDS model is fit using a modified EM algorithm, which requires computing the posterior over the latent variables. Due to the Poisson observation model an analytic form of this posterior is unavailable. Typically one replaces the exact posterior by its Laplace approximation, which accelerates model fitting but violates some assumptions of the EM algorithm, resulting in an approximate inference framework (Macke et al., 2011).

An application of PLDS to multi-electrode recordings from songbird auditory cortex by Buesing et al. (2017) revealed that responses are modulated by shared variability with a single latent state, a similar result to Ecker et al. (2014). Buesing et al. histologically traced the locations of the recording sites and found a spatial gradient in the strength of the latent states. Shared variability was stronger (i.e., neurons were more strongly coupled to the latent state) in deeper regions of auditory cortex. Interestingly, this strength was much weaker for certain stimulus classes than others, suggesting that deeper neurons selectively decouple from the latent state according to their stimulus preference. Other examples of dynamical systems-based latent factor models are given in Paninski et al. (2010), Buesing et al. (2012), Pfau et al. (2013), Semedo et al. (2014), Buesing et al. (2014), Archer et al. (2014), Kao et al. (2015), Gao et al. (2016), and Pandarinath et al. (2018b).

## 4. Generative Models for Calcium Imaging Data

### 4.1. Autoregressive Calcium Dynamics and Spike Deconvolution

The potential utility of large scale simultaneous neural recordings is constrained by our ability to make use of sophisticated techniques (such as latent factor methods) to analyse the data. While calcium imaging provides access to such large scale data, the models discussed so far assume that the data being analyzed is electrophysiological; i.e., that the neurons' responses are spike counts (for Poisson noise models) or firing rates (e.g., for Gaussian noise models). Their application to calcium imaging thus requires knowledge of how the optically recorded fluorescence signals are related to the underlying spiking activity. One approach to solving this problem involves constructing a generative statistical model where the spike counts are latent variables that are subsequently inferred from the fluorescence levels.

**Figure 4:**
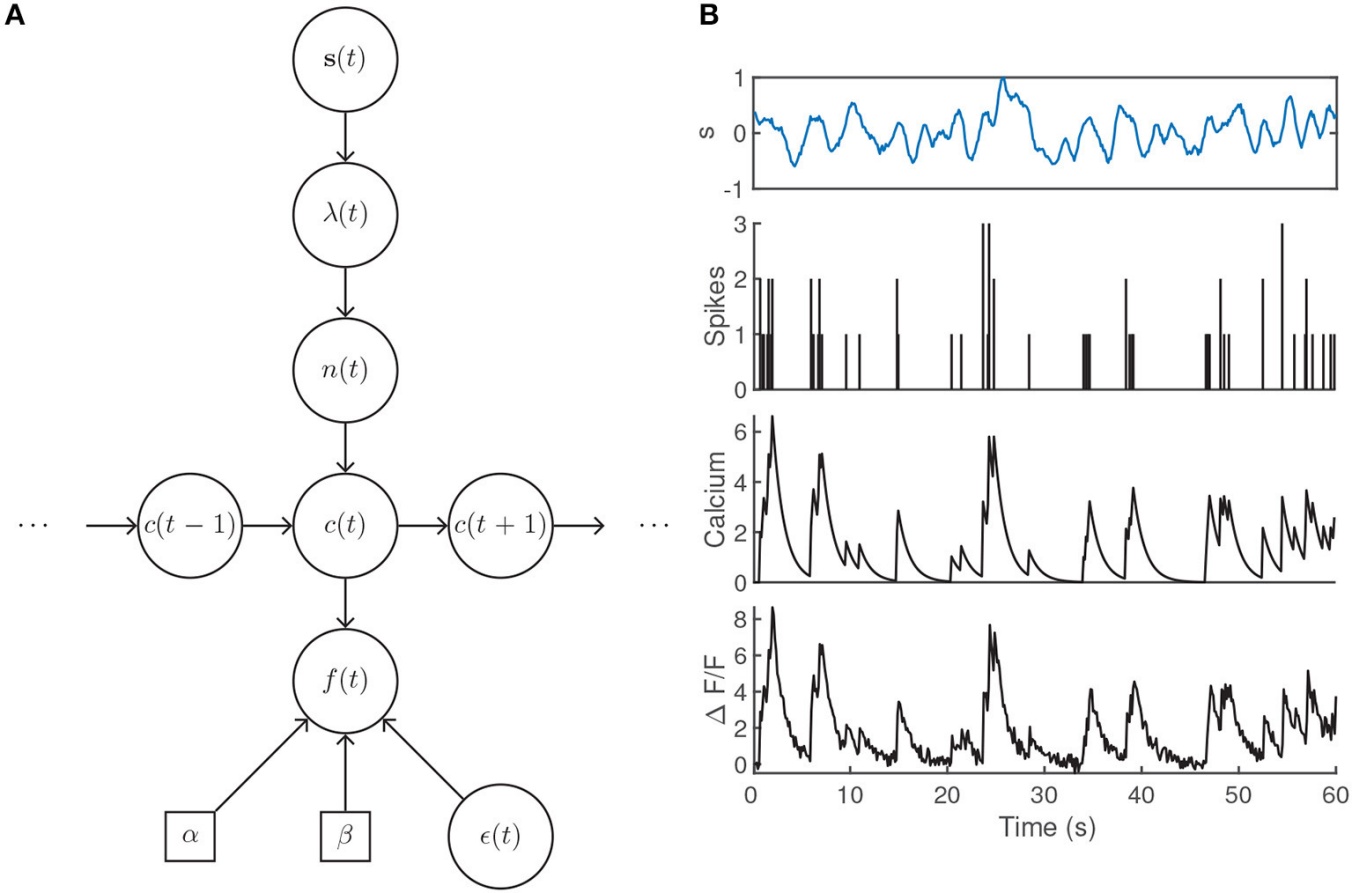
**(A)** Generative model architecture for fluorescent calcium imaging data. The stimulus sets the spiking intensity according to a neuron's receptive field. The resulting number of spikes within the timebin are drawn from a Poisson distribution, lead to rises in the intracellular calcium concentration, and are observed through noisy fluorescence levels. **(B)** Example fluorescence trace generated by a time-varying intensity function, first-order autoregressive calcium dynamics, and parameter values α = 1.25, β = 0.1, γ_1_ = 0.9, σ = 0.25 with an imaging rate of 8 Hz. The intensity λ(*t*) was determined by filtering the input stimulus *s*(*t*) by a Gaussian tuning curve centered at 0.25.

The presentation of a stimulus elicits a sequence of spikes across a population of neurons. For an individual neuron, we have assumed that the number of spikes within a time bin is sampled from a Poisson distribution with mean λ according to its particular receptive field. Each action potential is associated with a stereotypical rise and decay of the intracellular calcium concentration *c*(*t*), usually modeled by an autoregressive process of order *p* (suppressing initial conditions for clarity) (Vogelstein et al., 2009),

(23) $$\begin{array}{r} c\left(t\right)=\overset{p}{\underset{i=1}{\sum }}\gamma_{i}c\left(t-i\right)+n\left(t\right),\;n\left(t\right)\sim \text{Pois}\left(\lambda \right) \end{array}$$

where the Poisson-distributed random variable *n*(*t*) models the generation of spikes within a time bin and γ_1_, …, γ_*p*_ are the autoregressive coefficients that govern the rise and decay of the fluorescence levels. The observed fluorescence signal *f*(*t*) is then obtained by a linear transformation of the calcium levels with additive noise,

(24) $$\begin{array}{rc} f\left(t\right) & =\alpha c\left(t\right)+\beta +\epsilon \left(t\right),\;\epsilon \left(t\right)\sim \;N\left(0,\sigma^{2}\right) \end{array}$$

where α sets the scale of the fluorescence signal and *β* accounts for a baseline fluorescence that may be unique to the imaging set-up or due to specific biophysical properties of individual neurons. The Gaussian noise model is intended to encompass variability due to, e.g., light scattering and shot noise (Delaney et al., 2018). Note that this model does not set parameters for the scale or baseline of the calcium transient in Equation (23), as they are absorbed by α and *β* when the calcium is transformed to obtain the fluorescence (Vogelstein et al., 2009). An illustration of the generative model is given in Figures 4A,B.

For imaging systems where the rise time of the indicator is fast relative to the imaging rate a first-order autoregressive process is typically used, corresponding to an instantaneous rise and exponential decay of the calcium concentration. An autoregressive process of order 2 is used in situations where the rise time is slow relative to the imaging rate, in which case the calcium transient appears to approach its maximum amplitude gradually (Pnevmatikakis et al., 2016).

Models based on Equations (23, 24) have been used for spike train deconvolution (Vogelstein et al., 2009, 2010; Friedrich and Paninski, 2016; Pnevmatikakis et al., 2016). Let the vector $\theta =\left(\alpha ,\beta ,\lambda ,\sigma ,{\left\{\gamma_{i}\right\}}_{i=1}^{p}\right)$ denote the model parameters, and let **f** = (*f*(1), …, *f*(*T*))^⊤^ and **n** = (*n*(1), …, *n*(*T*))^⊤^. Following Bayes' rule, the maximum *a posteriori* estimate for the spike train is

(25) $$\begin{array}{r} \hat{\text{n}}=\underset{n\left(t\right)\in {\mathbb{N}}_{0}\;\forall t}{\mathrm{argmax}}p\left(\text{n}|\text{f},\theta \right)=\underset{n\left(t\right)\in {\mathbb{N}}_{0}\;\forall t}{\mathrm{argmax}}p\left(\text{f}|\text{n},\theta \right)p\left(\text{n}|\theta \right) \end{array}$$

where ℕ_0_ is the set of non-negative integers. Given the spike sequence **n**, the fluorescence levels *f*(*t*) are independent and depend only on the calcium concentration *c*(*t*), hence the likelihood factorizes as

(26) $$\begin{array}{r} p\left(\text{f}|\text{n},\theta \right)=\overset{T}{\underset{t=1}{\prod }}p\left(f\left(t\right)|c\left(t\right),\theta \right)=\overset{T}{\underset{t=1}{\prod }}N\left(f\left(t\right)|\alpha c\left(t\right)+\beta ,\sigma^{2}\right). \end{array}$$

Substituting Equation (26) into (25) and taking logarithms, the optimal sequence of spikes is then

(27) $$\begin{array}{c} \hat{n}=\underset{n(t)\in {\mathbb{N}}_{0}\;\forall t}{argmax}\overset{T}{\underset{t=1}{\sum^{\text{​}}}}\left\{-\frac{1}{2\sigma^{2}}{\left(f(t)-\alpha c(t)-\beta \right)}^{2}+n(t)\ln\;\lambda -\ln\;(n(t)!)\right\} \end{array}\begin{array}{c} . \end{array}$$

This is a difficult optimization problem because it requires searching through an infinite discrete space of spike trains. As noted in Vogelstein et al. (2010), even imposing an upper bound on the number of spikes within a frame yields an optimization problem with exponential computational complexity. One approach for overcoming this intractability involves approximating the Poisson distribution in Equation (25) by an exponential distribution, which leads to a concave objective function but with continuous estimates of $\hat{\text{n}}$ (Vogelstein et al., 2010). This approximation also allows for a time-varying intensity function λ(*t*), but does not explicitly model the transformation from stimulus to spiking intensity.

Runyan et al. (2017) applied a combination of the methods described in this review to study the timescales of population codes in cortex. 2-photon calcium imaging of auditory and posterior parietal cortices was performed while mice completed a sound localization task. The resulting fluorescence data was deconvolved according to the exponential-approximation approach described above to estimate firing rates (Vogelstein et al., 2010). They then fitted a GLM encoding model to populations from each cortical area that included coupling filters and various experimental and behavioral covariates. The fitted model was used in a decoding analysis that quantified the contribution of interneuronal coupling in the two cortical areas, and showed that stronger coupling was associated with population codes that had longer timescales. This provided evidence for a coding mechanism where tightly coupled populations of neurons prolonged the representation of stimuli through their sequential activation.

### 4.2. A Generalized Model for Calcium Dynamics

The calcium kinetics in Equation (23) are deterministic given the spike counts. In reality the concentration of calcium may be subject to many sources of variability, and analyses of some data sets may benefit from explicitly accounting for this noise. Vogelstein et al. (2009) modeled this by driving the calcium levels by both Bernoulli-distributed spikes and additive Gaussian noise,

(28) $$\begin{array}{rc} c\left(t\right) & =\gamma c\left(t-1\right)+n\left(t\right)+\xi \left(t\right) \end{array}$$

(29) $$\begin{array}{rc} n\left(t\right) & \sim \;\text{Bern}\left(p\left(t\right)\right) \end{array}$$

(30) $$\begin{array}{rc} \xi \left(t\right) & \sim \;N\left(0,\nu^{2}\right) \end{array}$$

where Bern(*p*(*t*)) is the Bernoulli distribution with time-dependent trial-success probability *p*(*t*), γ < 1 is an autoregressive coefficient, and ν^2^ is the calcium noise variance. A simplifying assumption in models based on Equations (23) and (24) is that spikes are generated independently of their spike history. However, the spike probability can be more generally modeled with a GLM (Vogelstein et al., 2009)

(31) $$\begin{array}{r} p\left(t\right)=1-\exp\left(-g\left({\text{w}}^{⊤}\text{s}\left(t\right)+{\text{J}}^{⊤}\text{h}\left(t\right)\right)\right) \end{array}$$

where *g* is a selected nonlinearity. Unlike the standard GLM structure of 8, the spike history term here takes the form $\text{h}\left(t\right)={\left(h_{1}\left(t\right),\ldots ,h_{L}\left(t\right)\right)}^{⊤}$, where each *h*_ℓ_ is an exponentially decaying refractory term that jumps following each spike

(32) $$\begin{array}{rc} h_{\ell }\left(t\right) & =\gamma_{h_{\ell }}h_{\ell }\left(t-1\right)+n\left(t\right)+\xi_{h_{\ell }}\left(t\right),\;\xi_{h_{\ell }}\left(t\right)\sim \;N\left(0,\nu_{h_{\ell }}^{2}\right). \end{array}$$

Finally, rather than a simple linear relationship between *f*(*t*) and *c*(*t*), Vogelstein et al. (2009) and Vogelstein et al. (2010) also consider saturating fluorescence levels using a nonlinear Hill function with dissociation constant *k*_*d*_

(33) $$\begin{array}{rc} f\left(t\right) & =\alpha \frac{c\left(t\right)}{c\left(t\right)\;+\;k_{d}}+\beta +\epsilon \left(t\right),\;\epsilon \left(t\right)\sim \;N\left(0,\sigma^{2}\right). \end{array}$$

Importantly, saturation of the fluorescence signal causes the spike-triggered fluorescence transients to become progressively smaller during a train of action potentials, and failure to account for this detail may limit the accuracy of spike deconvolution algorithms. The model defined by Equations (28–33) is fit using a sequential Monte Carlo method (Vogelstein et al., 2009). By including explicit stimulus and spike history filters, Vogelstein et al. (2009) could accurately infer spike times from fluorescence data with temporal superresolution; i.e., could identify when within an imaging frame each spike occurs. Some other example methods for spike deconvolution are based on compressed sensing (Pnevmatikakis and Paninski, 2013), fully Bayesian inference (Pnevmatikakis et al., 2013), and variational autoencoders (Speiser et al., 2017).

## 5. Discussion

Probabilistic modeling provides a practical, interpretable, and theoretically grounded framework for probing how networks of neurons process information. Many of the statistical models discussed in this review are abstract mathematical descriptions of how stimuli are related to patterns of neural activity. Often the mathematical operations that define the models do not necessarily attempt to align with real biological functions or behavior. Rather, such models are intended to serve as tools to uncover interpretable patterns and relationships that may not be detectable by other approaches. On the other hand, there are cases where the goal is to infer biophysical variables, as in e.g., models for calcium imaging data or for the anatomical architecture of a neural circuit, and then greater care must be taken to constrain the model by relevant physiological data (Paninski et al., 2007; Real et al., 2017; Latimer et al., 2018).

Recent advances in statistical models of spike train data have focused on incorporating more general nonlinear transformations of the latent state, including the use of neural networks (Gao et al., 2016; Pandarinath et al., 2018b) and GPs (Wu et al., 2017). This is in contrast to e.g., the FA and GPFA encoding models, where the mean spiking intensity of a neuron is obtained by a simple linear transformation of the latent state. Bayesian methods, such as latent factor modeling, are a powerful way to incorporate prior knowledge when making inferences about the behavior of a system. While GPFA places a smoothness prior on the evolution of latent factors to encourage some degree of temporal structure, other methods place priors on, e.g., network structure for connectivity inference (Linderman S. et al., 2016) and the latent states of a hidden Markov model with Poisson observations (Linderman S. W. et al., 2016).

Although there has been a rapid expansion in the number of models for extracting receptive fields, interneuronal coupling strengths, and latent structure from multivariate electrophysiological recordings, similar models for calcium imaging data are only beginning to emerge (Aitchison et al., 2017; Khan et al., 2018). A common approach for analysing calcium imaging data involves first deconvolving fluorescence traces and then fitting conventional models, but deconvolution methods only provide coarse estimates of firing rates. Spike trains obtained by highly optimized algorithms typically only agree with ground truth recordings with a correlation coefficient less than ~0.75, even with substantial training data, suggesting that there is an unavoidable loss of information associated with spike deconvolution (Pnevmatikakis et al., 2016; Berens et al., 2018). An advantage of GPFA over earlier methods for estimating trajectories of population activity is that it condenses the two stages of dimensionality reduction and smoothing into a single stage of posterior inference. Similarly, probabilistic analysis of calcium imaging data can have the two stages of deconvolution and model fitting merged into a single step by marginalizing over possible spike trains (Ganmor et al., 2016), mitigating some of the information loss accompanied by deconvolution. Neural encoding models for calcium imaging data that avoid an explicit intermediate step of spike inference are likely to be an important future development in this area (Aitchison et al., 2017).

Many studies consider the amplitude of an evoked calcium transient as a measure of a neuron's response. This has been widely used in zebrafish larvae, for which there has been significant interest in recent years. For example, 2-photon calcium imaging of the zebrafish optic tectum has led to new insights into the circuit architecture determining selectivity to size, location, and direction of motion (Del Bene et al., 2010; Gabriel et al., 2012; Grama and Engert, 2012; Nikolaou et al., 2012; Lowe et al., 2013; Preuss et al., 2014; Avitan et al., 2016; Abbas et al., 2017), and light-sheet microscopy has allowed for the creation of brain-wide functional circuit models for motor behavior driven by vision (Naumann et al., 2016) and thermosensation (Haesemeyer et al., 2018). Similar studies in the future provide further opportunities for model-based analyses.

The techniques described in this review were developed for spike train or calcium imaging data, but some approaches are broadly applicable across systems neuroscience. For instance, suitably adapted latent factor models have been successfully applied to recordings of the local field potential, where it was found that the activity of particular latent factors could discriminate vulnerability to stress-induced behavioral dysfunction in mouse models of major depressive disorder (Gallagher et al., 2017; Hultman et al., 2018).

As the scale of multi-neuron data continues to grow, the creation of new models and their associated fitting algorithms may be spurred more by efficiency and scalability considerations than the level of statistical detail they are able to extract from experimental data (Zoltowski and Pillow, 2018). In some cases the computational issues associated with neural data analysis are more profound than simply needing a larger computer cluster. Neuropixel electrode arrays (Jun et al., 2017), for example, are capable of recording from hundreds of channels simultaneously, and may put inference algorithms under strain if computational efficiency is not sufficiently addressed. When combined with fluorescent sensors of neural activity, optogenetic photostimulation grants the ability to manipulate neural circuits in real time, and models are now beginning to explicitly integrate the effect of photostimulation on calcium transients (Aitchison et al., 2017). Moreover, genetically encoded voltage indicators operate on a timescale of tens of milliseconds (Knöpfel et al., 2015), overcoming one of the principal drawbacks of calcium imaging; namely, the slow binding kinetics of the indicator relative to the timescale of action potential generation. Combining these emerging technologies with models designed to capture their associated generative processes thus promises to greatly improve our capacity to uncover how patterns of neural activity represent and process features of the external world.

## Author Contributions

All authors listed have made a substantial, direct and intellectual contribution to the work, and approved it for publication.

### Conflict of Interest Statement

The authors declare that the research was conducted in the absence of any commercial or financial relationships that could be construed as a potential conflict of interest.
